# Identification of Clinical Immunological Determinants in Asymptomatic VL and Post Kala-azar Dermal Leishmaniasis Patients

**Published:** 2018

**Authors:** Ashish Kumar SINGH, Vidya Nand Rabi DAS, Ajay AMIT, Manas R DIKHIT, Vijaya MAHANTESH, Akhilesh KUMAR, Raj Kishore PANDEY, Shyam NARYAN, Bipin K SINGH, Krishna PANDEY, Neena VERMA, Pradeep DAS, Sanjiva BIMAL

**Affiliations:** 1. Dept. of Immunology, Rajendra Memorial Research Institute of Medical Sciences, Patna, India; 2. Dept. of Pathology, Rajendra Memorial Research Institute of Medical Sciences, Patna, India; 3. Dept. of Microbiology, Rajendra Memorial Research Institute of Medical Sciences, Patna, India; 4. Dept. of Clinical Medicine, Rajendra Memorial Research Institute of Medical Sciences, Patna, India; 5. Dept. of Molecular Biology, Rajendra Memorial Research Institute of Medical Sciences, Patna, India

**Keywords:** Visceral leishmaniasis, Asymptomatic infection, Cytokine, Direct agglutination test, Interleukin-10, IFN-gamma, Tumor necrosis factor-alpha

## Abstract

**Background:**

Visceral Leishmaniasis (VL) caused by protozoa belonging to the genus *Leishmania,* usually have anthroponotic mode of transmission and is issue of great public health importance in Indian subcontinent. Asymptomatic cases of VL and PKDL are subject of keen interest to find their role in the transmission of VL in epidemic areas. We evaluated the immunological cytokine determinants expressed in most clinical suspects of asymptomatic VL and PKDL (IL-10, IFN-γ, and TNF-α).

**Methods:**

Eighty-four participants were included at RMRIMS, Patna, India in 2016–17 out of which 64 asymptomatic individual positive for rK-39, without sign and symptoms of VL; 15 PKDL patient’s with past history of VL and 5 endemic healthy subjects were recruited from VL endemic areas. DAT and quantitative assessment of plasma cytokines was determined from the blood samples collected in a plain and sodium-EDTA vacutainer respectively from the subjects.

**Results:**

The mean level of IL-10 in DAT_pos_^LOW^ of asymptomatic VL and PKDL was significantly higher than endemic healthy (*P*<0.05). The cytokine polarization index (IFN-γ versus IL-10) was significantly low in PKDL cases compared with asymptomatic VL cases in DAT_pos_^LOW^ titre (*P*<0.05). This index was low again but statistically not significant in PKDL than in asymptomatic VL when TNF-α was considered against IL-10. The ratio of IFN-γ: IL-10 and TNF-α: IL-10 was observed decreased both in asymptomatic VL and PKDL cases than in healthy from endemic areas.

**Conclusion:**

Collectively we surmise from our data that asymptomatic VL can also play an important role like PKDL in transmission of VL.

## Introduction

Visceral leishmaniasis (VL) is commonly known as kala-azar in Bihar State, India. The disease is transmitted to humans by the bite of the infected *Phlebotomine* sandfly (1). The *Leishmania donovani* parasite grows as an extracellular flagellated promastigote in the sandfly vector (2). In a mammalian host, the parasite is taken up by the macrophages where it multiples within its phagolysosomal vesicles.

Untreated visceral leishmaniasis is usually fatal (3). The most anti-leishmanial drugs (miltefosine, paromomycin, and amphotericin B) to treat patients are fraught with limitations such as cost, toxicity, and resistance. One problem in the fight against VL is the identification of potential risk groups in the community with a possible role in the transmission of VL. Important hot spots to track such risk group cases are asymptomatic cases and post-kala-azar dermal leishmaniasis (PKDL) patients residing in endemic areas. The transmission is anthroponotic, and *L. donovani* succeeds in surviving between successive VL epidemics (4).

Individuals who are PKDL with a history of VL co-exist in VL endemic areas during such epidemics with dermatosis with lesions in the form of hypo-pigmented macules, erythema, and nodules (5). Doubt exists regarding whether a PKDL patient can be a partner in the transmission of VL based on an exposed skin lesion, considered accessible to the sandfly vector. Many groups predict that 0.5% of PKDL patients will make VL epidemic. Clinical manifestations of PKDL depend on the types of immune response against parasites in the skin versus in visceral organs, where the immune reconstituted state is expected due to a previous VL episode.

There also remains the existence of asymptomatic patients of VL in the VL endemic community. Biomarkers are unavailable to know the proportion of illnesses that resolve after a short febrile illness or are entirely asymptomatic. Such information is of epidemiological significance, as only a few *L. donovani* infected patients convert into symptomatic VL at a given time (6, 7). As such, one might presume that subclinical infection is widespread in endemic areas during VL epidemics (8). Taken together, both asymptomatic and PKDL patients are hot spots in terms of understanding their role and probable intensity in the transmission of VL in endemic areas, and we urgently need biomarkers to identify these cases during epidemics of VL (6, 8).

Diagnosis in VL patients is confirmed by the presence of parasites by culture or the microscopical examination of bone marrow and splenic smears of parasite DNA in PCR-based assays (9). Complete reliance on this approach is likely to miss early cases, and it will make a tracking system difficult for early asymptomatic cases. Alternatively, PKDL is confirmed microscopically by the presence of the parasite in a slit/biopsy sample or by DNA in PCR-based techniques (10).

Antibody detection methods, in a serodiagnostic approach, are non-invasive, sensitive, relatively simple, and cost-effective (7, 11–13). Direct agglutination test (DAT) has been known as a validated method for serodiagnosis and seroepidemiological studies of VL and PKDL among human in many parts of the world including India and Iran (14–17).

Many other less invasive methods are recommended for the rapid detection of such early cases in endemic areas. These include an improved DAT using freeze-dried antigen (18, 19), a rapid immune-chromatographic test based on recombinant 39-amino acid repeat antigen conserved in the kinesin region of *L. chagasi* and *L. donovani* (rK-39 strip test) (20), a similar strip test based on a recombinant 26 kDa protein (rK-26 strip test) (21), rKE16 (22), and a latex agglutination based on the direction of heat stable carbohydrate *Leishmania* antigen in the urine of a VL patient (KAtex) (23, 24). Such tests are able to track hot spots, but they may not indicate the actual factors for the emergence of asymptomatic VL or PKDL cases and their role as determinants for the transmission of VL.

The pathophysiological situation of a VL and PKDL patient is determined by cytokines produced during the cellular immune response to identify the subsequent resistance or susceptibility of the patient to the disease (25, 26). In the absence of a Th1-type immune response (IL-2, IFN-γ, TNF-α), patients present with pathogenicity and problems of resistance occur only thereafter (27). This concept is valid as, when genetic ablation of IL-6 and IL-10 occurs, a strong Th1-immune response is mounted that cures all forms of leishmaniasis (27–29).

In the present study, we evaluated the immunological cytokine determinants expressed in most clinical suspects of early VL (asymptomatic VL) and PKDL (IL-10, IFN-γ, and TNF-α) in predicting the transmission of VL in endemic areas.

## Materials and Methods

### Subjects and Sample

Overall, 84 participants in 2016–17 were included and comprised of 64 asymptomatic individuals from the endemic area of Bihar, India, who were positive for rK-39 (InBIOS, USA) without signs and symptoms of VL; 15 recently clinically diagnosed PKDL patients (1–3 months) with a past history of VL at the Rajendra Memorial Research Institute of Medical Sciences (RMRIMS), Patna, India; and 5 endemic healthy subjects aged ≥1 yr.

After obtaining informed consent, whole blood (5 ml) was collected by venipuncture in a plain and sodium-EDTA vacutainer (BD Biosciences, USA) from each study subject. The serum was extracted from the plain vacutainer and utilized for both rK-39 and the DAT test while plasma was extracted from the sodium-EDTA vacutainer for quantitative assessment of cytokines (through sandwich ELISA).

The protocol of this study was in accordance with the recommendations outlined in the Helsinki Declaration, and ethical approval was obtained from the Ethical Review Committee of Human Studies, RMRIMS, Patna, India.

### rK-39 Strip Test

Kalazar Detect^TM^, a commercial version of the immunochromatographic strip test based on the rK-39 antigen, was purchased from InBios International (Seattle, WA) and used according to the manufacturer’s instructions. For each test, 5–10 μl serum was placed on the absorbent pad on the nitrocellulose membrane followed by two or three drops of chase buffer. A positive result was indicated by the appearance of two pink lines after 5 min (one control and one test).

### Direct Agglutination Test (DAT)

The antigen for the DAT was prepared and a test performed (30). Briefly, a serial doubling dilution of each serum was prepared in normal saline supplemented with 0.2% (w/v) gelatin and 0.78% (v/v) 2-mercaptoethanol in the V-shaped wells of a 96-well microtitre plate (Nunc, Roskilde, Denmark). The first well of the plate was an antigen control containing 50 μl of diluent and no serum, while each of the other wells contained 50 ml diluted test serum. Then 50 μl of a suspension of fixed and stained *L. donovani* promastigotes (7.5×10^7^/ml) were added to each well. The plate was gently swirled on a level surface for 30 sec. Then it was covered and incubated at 18–22 °C for 18 h. The test results were read visually, with titres expressed as the highest dilutions of serum giving agglutination (50% mesh and 50% bead in the end-point titre). Only samples that agglutinated at a dilution of 1:800 were considered seropositive.

### Cytokine Produced During Cellular Immune Response

Plasma cytokine (IFN-γ, IL-10 and TNF-α) were determined by EMD Millipore, USA, according to the manufacturer’s instructions. The detection limits were 5.6 pg/mL, 2 pg/mL, and 3.5 pg/mL for IFN-γ, IL-10, and TNF-α, respectively. All samples were run in triplicate wells. The color intensity was read at a wavelength of 450 nanometers in an ELISA reader (Bio-Rad).

### Statistical Analysis

A student t-test was used to determine the difference between the groups. Statistical analysis was performed using GraphPad Prism 5 (USA software). All the data expressed as Mean±SE and *P*≤0.05 were considered statistically significant for the analysis.

## Results

**Fig.1:**
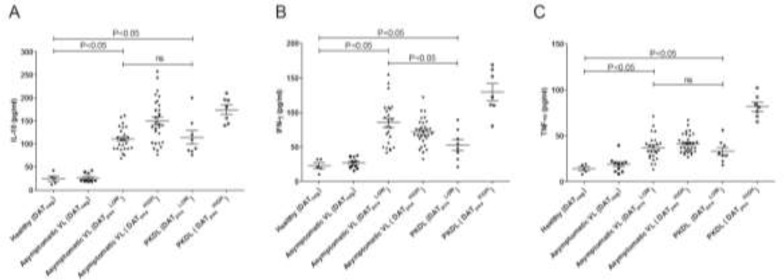
Comparison of various immunological factors with asymptomatic visceral leishmaniasis (Asymptomatic VL) and post-kala-azar dermal leishmaniasis (PKDL) patients in comparison to endemic healthy control. Total cytokine production (pg/mL) (a) IL-10 (b) IFN-γ and (c) TNF-α from Healthy, Asymptomatic and PKDL subjects. Student t-test; ns, non-significant; *P*≤0.05, significant

Out of the 84 enrolled subjects, 79 were initially observed as rK-39 positive. All five healthy subjects gave a negative result in the rK-39 test. For confirmation, a serodiagnostic DAT was performed on the 79 subjects. Based on the anti-*Leishmania* antibody titre observed in the DAT, the subjects were divided into three groups: those that were DAT negative (<1:800) (DAT_neg_), a positive DAT at a low titre range (1:1600–1:6400) (DAT_pos_^LOW^), and a positive DAT with a high titre range (>1:6400–1:25600) (DAT_pos_^HIGH^). Based on such categorization, we identified 10 rK-39 positive asymptomatic cases under DAT_neg_, 24 asymptomatic cases under the DAT pos ^LOW^ titre range, and 30 asymptomatic cases under the DAT_pos_^HIGH^ titre range. In 15 PKDL patients, 8 were under DAT _pos_^LOW^ and 7 were under DAT_pos_^HIGH^ dilution. All healthy subjects gave a negative result in the DAT assay (<1:800). This test (DAT) is the gold standard for serodiagnosis and also a high range of antibody titre may indicate more exposure to *L. donovani*, the patients were referred to as DAT positive low or high. The mean level of IL-10 in DAT pos ^LOW^ of asymptomatic VL and PKDL was comparable. Both groups produced significantly higher IL-10 than was observed in healthy subjects (*P*<0.01). In this category (DAT_pos_^LOW^), the IFN-γ levels in asymptomatic VL patients were higher than in PKDL (*P*<0.01) patients (Table 1, Fig. 1).

**Table 1:** Comparison of various immunological factors with asymptomatic visceral leishmaniasis (Asymptomatic VL) and post-kala-azar dermal leishmaniasis patients (PKDL)

***Subjects***	***DAT***	***N***	***Cytokine (pg/ml)***		
	**Anti-*Leishmania* titre**		**IL-10**	**IFN-γ**	**TNF-α**
Healthy	<1:800	5	24.00±5.32	22.80±4.19	14.00±2.07
Asymptomatic VL	<1:800	10	25.69±3.015	26.74±2.53	18.66±2.79
	1:1600–1:6400	24	110.50±5.33	84.95±6.54	37.02±2.85
	>1:6400	30	150.30±8.62	71.31±3.58	40.96±1.87
PKDL	<1:800	0	Nil	Nil	Nil
	1:1600–1:6400	8	113.80±14.71	52.88±7.88	33.03±4.32
	>1:6400	7	174.30±10.46	129.40±12.30	81.57±4.76

**Fig. 2:**
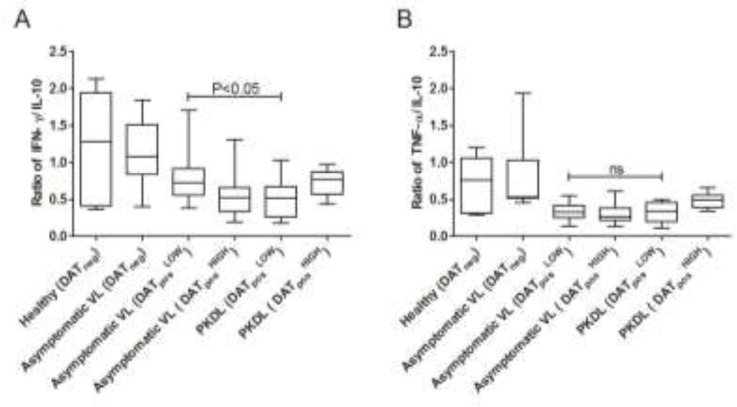
Cytokine polarization index in asymptomatic visceral leishmaniasis (Asymptomatic VL) and post-kalaazar dermal leishmaniasis (PKDL) patients. Cytokine ratio (a) IFN-γ / IL-10 (b) TNF-α / IL-10 of Healthy, Asymptomatic and PKDL subjects. Student t-test; ns, non-significant; *P*≤0.05, significant

The cytokine polarization index (IFN-γ versus IL-10) was significantly low in PKDL cases compared with asymptomatic VL cases in DAT_pos_^LOW^ titre (*P*<0.05). This index was again lower in PKDL than in asymptomatic VL when TNF-α was considered against IL-10 in DAT_pos_^LOW^ titre, but we observed statistically not significant (*P*>0.05) (Fig. 2). PKDL cases, even at DAT_pos_^LOW^ titre, could be more vulnerable and critical for the transmission of VL. In DAT_pos_^HIGH^ titre cases, the mean cytokine polarization index for IFN-γ versus IL-10 (Fig. 2) was high, but it was statistically not significant in PKDL patients compared with asymptomatic VL subjects. A greater defect was observed in the activity pattern of mean TNF-α compared with IFN-γ in PKDL patients with DAT_pos_^HIGH^ titre.

## Discussion

As the anti-*Leishmania* titre increased in the DAT, we observed a considerable increase in the production of IL-10. This trend becomes equally effective both in asymptomatic VL cases (identified initially based on rK-39) and in PKDL cases (31). Initially, we identified rK-39 positive asymptomatic VL and PKDL cases and categorized these subjects on the basis of DAT ^LOW^ and DAT ^HIGH^ titre result. The purpose of such categorization was meant to identify major immunological determinants in asymptomatic VL and PKDL cases and to relate them as possible factors in the transmission of VL. PKDL cases associated with a greater yield at IL-10, as was reported earlier as an important immunological marker observed in the skin and plasma of Sudanese PKDL patients (32). The high incidence of drug resistance may be due to anthroponotic transmission via PKDL (33). Therefore, there is no doubt regarding the influence of PKDL patients as a possible reservoir of VL.

The present study suggested that VL cases in their asymptomatic stage and PKDL diagnosed by rK-39 and observed with high antibody DAT titre can be an early immunological determinant of VL reservoir in the endemic area. IL-10 is associated with the progression of VL by obstructing the Anti-*Leishmania* function of macrophages and the regulating effects of protective IFN-γ (27, 29, 34). We also looked for evidence of the involvement of other immune modulators in VL transmission. We especially looked for the role of IFN-γ and TNF-α during IL-10 expression in both groups of patients. Even asymptomatic VL patients could play a paramount role in the transmission cycle. We observed that when a healthy individual is exposed and anti-*Leishmania* antibodies appear in asymptomatic patients above the cut-off, but at slightly higher dilution (1:1600–1:6400), even the IL-10 produced in comparatively lower concentration compared with PKDL suppresses the effect of IFN-γ and TNF-α. The ratio of IFN-γ: IL-10 and TNF-α: IL-10 was observed as decreased both in asymptomatic VL and PKDL cases than in healthy from endemic areas. Both IFN-γ and TNF-α are involved in several anti-*Leishmania* activities such as the production of free radicals (iNOS and ROS) and help phagocytic cells to clear the parasite (35). In contrast, IL-10 inhibits the effect of pro-inflammatory cytokines which often results in severe disseminated forms of infection (35–37). Therefore, *Leishmania* parasites in asymptomatic VL cases and PKDL patients shifted the immune response more towards IL-10 and made them more susceptible to disease (35).

## Conclusion

The asymptomatic VL can also play an important role like PKDL in transmission of VL. However, further investigation delineating more immunological determinants may lead to better immune intervention to restrict the transmission of VL in endemic areas.
